# Resveratrol-Based Multivitamin Supplement Increases Sperm Concentration and Motility in Idiopathic Male Infertility: A Pilot Clinical Study

**DOI:** 10.3390/jcm9124017

**Published:** 2020-12-11

**Authors:** Ester Illiano, Francesco Trama, Alessandro Zucchi, Rossana Giulietta Iannitti, Bernard Fioretti, Elisabetta Costantini

**Affiliations:** 1 Andrologycal and Urogynecological Clinic, Santa Maria Terni Hospital, University of Perugia, 05100 Terni, Italy; ester.illiano@inwind.it (E.I.); elisabetta.costantin@unipg.it (E.C.); 2 Urological Clinic, University of Pisa, 56125 Pisa, Italy; zucchi.urologia@gmail.com; 3 Department of Chemistry, Biology and Biotechnologies, University of Perugia, Via Elce di Sotto 8, 06132 Perugia, Italy; r.iannitti@srfarmaceutici.com (R.G.I.); bernard.fioretti@unipg.it (B.F.)

**Keywords:** resveratrol, male infertility, mitochondrial activity

## Abstract

Background. It is known that a multitude of factors may lead to male factor infertility, but still, in the majority of cases, the cause remains largely idiopathic, reflecting poor understanding of the basic process of spermatogenesis and the mechanisms involved. Resveratrol is a polyphenol compound that displays several cellular aspects mainly associated with SIRT1-pathway activation and promotion of mitochondrial enhancer activities. In several animal models, resveratrol has shown positive effects on mitochondria and membrane potential. This could explain effects on sperm concentration and motility. The aim of this study is to evaluate the effects on the semen parameters of GENANTE^®^, a multivitamin supplement containing 150 mg of resveratrol/day, in patients with idiopathic infertility. Methods. This was a prospective single center clinical study. Twenty patients took a multivitamin supplement based on 150 mg of resveratrol (GENANTE^®^), in the form of an oral tablet every 12 h, and were followed up at 1, 3, and 6 months after treatment. Pre- and post-treatment evaluation included history, clinical examination, semen analysis, hormonal determinations, and scrotal and prostatic ultrasound. Results. Our preliminary pilot study demonstrated that the multivitamin supplement based on resveratrol improves sperm motility (48.3% ± 13.8 vs. 59.0% ± 12.8, *p* = 0.0001) and concentration (22.6 × 10^6^/m^L^ ± 9.5 vs. 25.7 × 10^6^/mL ± 8.1, *p* = 0.0001) after 3 and 6 months of treatment in men with idiopathic infertility. Conclusion. Our data suggest that targeting the metabolic and energetic pathways involved in spermatogenesis and mitochondrial activity could lead to potential effects and counteract subfertility/infertility in men through a mitochondria dynamics mechanism. Trial registration number: ClinicalTrials.gov registration identifier: NCT03864198, registered on 1 January 2019.

## 1. Introduction

Infertility is defined as the inability to achieve spontaneous pregnancy after at least one year of regular, unprotected sex [1]. Infertility affects 15–20% of couples [2]. A male factor is estimated to be present in approximately 50% of cases, with sole responsibility in 30% of cases and a co-contributing female factor in 20% of cases [3]. Male infertility may include the abnormal semen parameters (oligozoospermia, asthenozoospermia, teratozoospermia) or a combination of all three (oligo-astheno-teratozoospermia), or azoospermia [4]. The causes of male infertility can be divided into four main areas (endocrine and systemic disorders, primary testicular defects in spermatogenesis, sperm transport disorders), including idiopathic infertility which affects up to 25% of patients [4]. Idiopathic male infertility is clinically diagnosed after excluding all other known causes of infertility.

Semen quality has often been used as an indirect measure of male infertility. This includes examination of sperm count, motility, and morphology. The majority, 80%, of altered parameters accounting for low sperm concentration are associated with a decrease in sperm motility (asthenozoospermia) and spermatozoa with normal morphology [5]. Whether there is a deterioration of semen quantity or quality is controversial [6,7]. However, there seems to be a clear trend toward a decline in sperm quality in our society [8]. There have been several explanations for this phenomenon. They include environmental stress, a modern lifestyle, infection, and/or chemicals that may alter the endocrine system. The result is a steady decline in male reproductive potential [9].

It is also known that in most infertile men who have abnormalities in sperm count, morphology, and/or motility, there is no identifiable cause [4]. Numerous nutritional [10,11,12,13,14] and medical interventions (hormonal therapies that modulate the hypothalamic–pituitary–testicular axis) have been used to treat male idiopathic infertility [2]. However, the management of these patients remains challenging. This is primarily due to the large numbers of various products and the conflicting evidence from individual trials. Most of the studies on infertility treatment, both in vitro and in vivo, have focused on oxidative stress mechanisms [15,16,17]. The oxidative stress mechanism could cause: lipid peroxidation, with alteration of membrane fluidity and permeability, which results in a decrease of sperm motility and in a reduction of sperm interaction with the oocyte; protein modification which causes a reduction of ATP production; or sperm DNA fragmentation [16].

In particular, the focus has been on increasing seminal antioxidant capacity, reducing the production of reactive oxygen species (ROS), stabilizing sperm chromatin (through zinc-based molecules), and inducing sperm capacitation (functional maturation of the spermatozoa). By contrast, few studies have centered on other mechanisms involved in metabolism, mitochondrial energy, and metabolism/mitochondrial function, which has recently emerged as one of the important factors in infertility physiopathology [18,19].

Resveratrol, trans-3,5,4′-trihydroxystilbene, is a polyphenol compound present in grapes, peanuts, berries, and wine [13]. It is a phytoalexin whose biological function is to protect the plant in case of parasitic attack or environmental stress [14]. Scientific reports have identified a wide variety of characteristics of this molecule. This includes anti-inflammatory, cardioprotective, anticancer, antimicrobial, antiaging, and antioxidant effects [20]. Resveratrol is also the most potent natural compound that activates sirtuin 1 (SIRT 1), the most-conserved mammalian NAD+-dependent protein, and a member of the family of sirtuins, which may account for its many metabolic benefits in humans [20].

Recent studies in animal models have demonstrated that resveratrol has a positive effect on the hypothalamic–pituitary–gonad axis, as well as blood testosterone levels, sperm production, and sperm motility [21,22]. Furthermore, resveratrol may decrease germ cell apoptosis [18,23]. An animal study with resveratrol and lycopene in post-thaw bull sperm demonstrated that resveratrol offered high mitochondrial activity, sperm motility, and DNA integrity [24]. It improved DNA integrity and sperm parameters in streptozotocin-nicotinamide-induced type 2 diabetic rats [25]. In vitro treatment with 15µM/mL of resveratrol on human sperm revealed that it has the capability to counteract the detrimental effects of benzo-α-pyrene exposure on sperm motility, abnormal chromatin compactness, lipid peroxidation, and mitochondrial superoxide [26]. Resveratrol can protect the quality of the mitochondria and increase its membrane potential [27], an effect that possibly accounts for the positive effect on sperm motility and explains the improvement of total and progressive sperm motility.

Despite these insights, the effect of resveratrol supplementation on male infertility has not yet been explored.

The aim of this study was to evaluate the effects of a nutraceutical based on resveratrol, (GENANTE^®^, a twice-a-day multivitamin supplement containing 150 mg of resveratrol, vitamin D, B6, B12, and folic acid) on the semen parameters of patients with idiopathic infertility. The primary outcome was to evaluate the semen parameters before and after 1, 3, and 6 months of treatment.

## 2. Material and Methods

This was a prospective single center study. The project was accepted by the local ethics committee and registered on clinicaltrials.gov (NCT03864198). We included idiopathic infertile male patients. The inclusion criteria were as follows: age 18–50 years and patients with oligozoospermia (<5 million spermatozoa/mL) and/or with asthenozoospermia (<32% progressive motile spermatozoa) e/o in accordance with WHO criteria [28]. The following patients were excluded: patients with azoospermia; patients who smoked and/or used drugs, or had taken drugs with proven fertility toxicity; patients with a history of consumption of alcohol; those who were exposed to any environmental or occupational toxic substances, including radiation, intensive cell-phone use or heat (patients who claimed to have a mobile phone in their front pocket for at least 5 h, for at least 10 min/h); patients who had epididymitis, epididymo-orchitis or orchitis secondary to mumps, bacterial infections, or sexually transmitted diseases; patients with a history of cryptorchidism, previous testicular torsion, genitourinary anomalies, alterations of the epididymis or deferens, and/or inguinal surgery; patients with hormonal alterations. The reason for exclusion was the causal relationship of these conditions with the deterioration of fertility [29,30,31].

The pretreatment evaluation included a patient history; clinical examination; semen analysis; hormonal determination (follicle-stimulating hormone (FSH), luteinizing hormone (LH), total testosterone, estradiol, prolactin, and 25-OH-Vitamin D3); a scrotal ultrasound to exclude signs of obstruction (e.g., dilatation of rete testis, enlarged epididymis with cystic lesions, or absent vas deferens), signs of testicular dysgenesis (e.g., non-homogeneous testicular architecture and microcalcifications), or testis tumors; and a prostatic transrectal ultrasound to exclude distal obstruction and any male accessory gland infection [32,33].

Hormonal assessments were performed in the same laboratory, and their evaluation was based on reference ranges for normal men provided by the laboratory measuring the samples.

In our laboratory, normal ranges were: prolactin 3.46–19.40 ng/mL; FSH 0.95–11.95 mUI/mL, LH 1.14–8.75 mUI/mL; total testosterone 10–20 years: 18.5–48.3 pg /mL, 20–30 years: 19.5–51.7 pg/mL, 30–50 years: 16.1–47.9 pg/mL, >50 years 12.1–39.6 pg/mL; estradiol 11–44 pg/mL; 25-OH-Vitamin D3 < 20 ng/mL deficiency, 20–29 ng/mL insufficient, 30–100 ng/mL sufficient. All assessments were certified (certified quality system UNI EN ISO 9001:2015).

Laboratory testing of testosterone to determine diurnal variation was carried out with two morning samples (7.00 a.m. and 11.00 a.m.). Prolactin levels are influenced by several factors, such as pick-up time (the prolactin secretion has a circadian rhythm, with high levels in the night and low during the day). However, since it was not possible to do so during sleep, dosage was carried out during the day, with three samples taken at intervals of 10–30 min. Estradiol, FSH, and LH were assayed by one sample.

Scrotal and transrectal ultrasounds were performed by the same urologist.

Patients were prescribed a multivitamin supplement (trademark GENANTE^®^, S&R Farmaceutici S.p.A. Bastia Italy). It consisted of REVIFAST^®^ (160 mg), trans-resveratrol (102 mg), Vitamin B6 (1.4 mg), Vitamin B 12 (2.5 mg), Vitamin D (25 mcg), and Extrafolate S^®^ (400 mcg). They received an oral tablet every 12 h, for a total of 2 tablets a day, for a daily consumption of 150 mg of resveratrol.

REVIFAST^®^ is the trade name of a new ingredient based on resveratrol that is supported by a magnesium hydroxide matrix at the concentration of 30% *w*/*w* [34]. Resveratrol is known to have low solubility in water and good membrane permeability and accordingly is a class 2 molecule by pharmaceutical classification, and therefore, it is poorly bioavailable [34]. Furthermore, it is known to have a fast metabolism that converts it to glucuronide and sulfate compounds.

Extrafolate S^®^ is the biologically active form of folic acid. This allows it to bypass any polymorphisms of the tetrahydrofolate methylene (MTHFR) gene reductases responsible for reduced enzyme activity of MTHFR.

All patients were followed at 1, 3, and 6 months after treatment with Genante© using the same pretreatment flow chart. Scrotal and transrectal ultrasounds were performed during follow up to rule out de novo pathologies.

All patients signed an informed consent form explaining the nature of the study and the possibility of treatment failure.

### Statistical Analysis

With the enrolment of 20 patients, *p* = 0.05, and the use of the χ^2^ test, the study was estimated to have an 80% power rejection of the null hypothesis that Genante^®^ does not change the seminal parameters in infertile patients. The power of the study was calculated using PS Power and Sample Size ver. 3.0, 2009. Continuous variables were presented as median values, and categoric data were presented as absolute or relative frequencies. Statistical analysis was performed using the Wilcoxon Signed Rank test to compare the variables, and the χ^2^ test and McNemar test for categorical data. All calculations were performed using IBM-SPSS^®^ version 22.0 (IBM Corp., Armonk, New York, NY, USA, 2013). A two-sided *p*-value < 0.05 was considered significant.

## 3. Results

Between January 2019 and June 2019, 20 patients, with idiopathic infertility according to WHO criteria, underwent treatment with Genante^®^ in our tertiary urological center. The demographic and clinical characteristics of the included patients are shown in Table 1.

Half of the patients were married; the other half had stable relationships, but they were not married. The most frequent sperm abnormality among the included patients was oligoasthenozoospermia (95%). At pretreatment, all patients had normal transrectal prostatic ultrasounds and scrotal ultrasounds.

**Figure 1:**
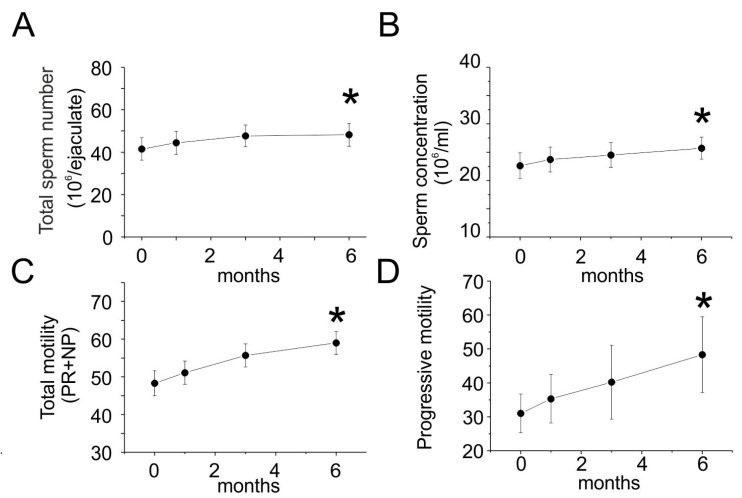
The laboratory assessment after six months of treatment. Data presented as ± SD. Significant differences between control and treated cells are denoted as * (*p* ≤ 0.05). (**A**): change in the total number of spermatozoa in six months. (**B**): change in the sperm concentration in six months. (**C**): change in the total motility (PR+NP) in six months. (**D**): Change in progressive motility in six months.

After six months of treatment, the laboratory assessment showed a statistically significant improvement in total sperm count (41.5 × 10^6^/ejaculat*e* ± 22.1 vs. 48.2 × 10^6^/ejaculate ± 22.4, *p* = 0.002), sperm concentration (22.6 × 10^6^/mL ± 9.5 vs. 25.710^6^/mL ± 8.1, *p* = 0.0001), total motility (48.3 ± % ± 13.8 vs. 59.0 ± % ± 12.8, *p* = 0.0001), and progressive motility (20% vs. 90%, *p* = 0.0001) (Table 2 and Figure 1).

The improvement of all the parameters was recorded previously at 1 and 3 months, with a progressive increase over time (Table 2). Sperm morphology, volume, and PH were not changed after treatment. The hormonal determinations were rather stable during follow-up (Table 3). The scrotal and transrectal prostate ultrasounds continued to be in the normal range.

## 4. Discussion

It is known that a multitude of factors may lead to male factor infertility; however, in the majority of cases, the cause remains largely idiopathic, which reflects a poor understanding of the basic process of spermatogenesis and the mechanisms involved [19].

Sperm density may be influenced more by many nutraceuticals or micronutrients, such as vitamin D, B and folic acid, while limited nutrients influence sperm motility. Folic acid is known to increase sperm density significantly following three months of folic acid supplementation to patients with oligospermia or asthenospermia [35]. By contrast, no statistical correlations were found between seminal plasma vitamin B6 level and sperm motility, sperm count, or semen volume [36]. Folate and B12 are not correlated with any semen parameters [37] but are known to modulate homocysteine. Vitamin D demonstrates a direct and positive relationship between serum vitamin D level and overall semen quality, male reproductive potential, and testosterone levels [38] and may enhance sperm motility by promoting the synthesis of ATP through the cAMP/PKA pathway [39]. However, in the literature, controversial data exist regarding vitamin D status and reproductive parameters [40], and thus, the role of vitamin D in male fertility is still debated.

Our study shows that a resveratrol-based multivitamin treatment increases the concentration of sperm cells and motility, which suggests an improvement in both the spermatogenesis process and fertilization potential. The process of spermatogenesis comprises the differentiation of the primordial germ cells into spermatogonia, followed by the production of primary and secondary spermatocytes, spermatids, and ultimately highly specialized mature spermatozoa [41]. Sertoli cells (SCs) play a key role in spermatogenesis by providing the essential physical support for developing germ cells and ensuring that they have the appropriate nutrients, energy sources, hormones, and growth factors.

In fact, spermatogenesis is highly dependent on energy metabolism [42] and glycolytic metabolism, as the lactate produced by the Sertoli cells is the major substrate of germ cells [43]. The mitochondria of the isolated germ cells produce ATP potentially, at close to a maximal rate. Spermatogenesis, therefore, may be extremely sensitive to compounds which interfere with mitochondrial energy metabolism and respiratory control. Any alteration in the regulation of these cells’ metabolic behavior may compromise the normal development of spermatogenesis and, consequently, male fertility [42,43]. It has been proposed that mitochondria also play a role in this degenerative process of the sperm, thereby assuring that good quality meiotic products enter the process of spermatogenesis to yield quality mature sperm [19]. Interestingly, resveratrol was demonstrated to increase the mitochondrial number (mitogenesis) and activity (ATP concentration) in several cell types, such as muscle cells [20] and granulosa cells [44]. Resveratrol improves mitochondrial function by activating sirtuin 1 (SIRT1) [20]. SIRT 1 is related to multiple age-associated diseases due to its capacity to deacetylate histones and non-histone proteins, such as tumor protein p53 (p53), kB-gene binding nuclear factor (NF-κB), heat shock factor 1 (HSF1), forkhead box transcription factor, class O (FOXOs), and peroxisome proliferator-activated receptor γ (PPARγ) coactivator-1 (PGC-1). Thus, it can regulate the cell’s biology, metabolism, and fate at various levels [45]. Therefore, sirtuins play an important role in a broad spectrum of biological processes. Their regulation of glycolytic metabolism and mitochondrial energy metabolism–respiratory control not only increases their physiological relevance to the testicular environment [46]; however, it also suggests that these metabolisms control sperm functionality and thus male reproductive health. Further studies are required to conclusively demonstrate if this effect on males occurs in male gamete cells during resveratrol treatment and if it is dependent on the SIRT-1 pathway.

Mitochondrial activity is also critical for mature sperm cells as it is correlated with sperm motility, an important factor for the penetration of the cumulus cells and zona pellucida of the oocyte [23]. In mature sperm, mitochondria cover the axosome and the associated dense fibers of the midpiece, via oxidative phosphorylation (OXPHOS), which increases the production of adenosine triphosphate (ATP) [18]. The inner mitochondrial membrane includes several complexes (electron transfer chain, ETC), which transport electrons derived from the oxidation of dihydroflavine–adenine dinucleotide (FADH2) and the nicotinamide adenine dinucleotide (NADH). In this process, an osmotic proton gradient is generated across the inner mitochondrial membrane and is subsequently used by the ATP synthase to phosphorylate adenosine diphosphate (ADP) to ATP. OXPHOS-derived ATP seems to be important for sperm motility [18]. The expression of several sperm mitochondrial proteins, including ETC complexes [47], may be altered in asthenozoospermic patients [23,48]. Many different ETC inhibitors have been shown to negatively affect sperm motility [49], both in humans [49,50] and in engineered mice [51]. Interestingly, there is a strong correlation with inner mitochondrial membrane potential (DP) and spermatic motility. In this context, the increase of motility observed in our study due to resveratrol treatment may be associated with this incremented mitochondrial membrane potential and metabolic activity. Mitochondria, in the mid-piece of mature mammalian spermatozoon, are fundamental for the creation of energy which is useful for sperm movement [19], and mtDNA genetic defects may compromise sperm physiology, and, in particular, motility [19]. Multiple mtDNA rearrangements are associated with decreased sperm motility [52]. In addition, the reduction of energy production may induce meiotic arrest during spermatogenesis [19].

It is unlikely that this is an increase in the expected number of mitochondria as the number of mitochondria is highly dependent on the neck volume of sperm cells. The beneficial effect of resveratrol agrees with the inverse correlation of mtDNA; in fact, mtDNA would be advantageous to developing spermatozoa [19], but not in mature sperm cells. Oligozoospermic and asthenozoospermic men have sperm containing significantly elevated levels of mtDNA [53], prompting the hypothesis of an optimal threshold for spermatozoa. Since the energy metabolism is important in both spermatogenesis and oxidative phosphorylation, it has been suggested as a determinant of sperm motility and functionality.

We are aware that this study may have some limitations. One is related to its small sample size and study design as a prospective clinical study. The second is the lack of evaluation of the impact of redox status in the effects observed since mitochondrial ETC promotes the production of ROS [18]. Balanced ROS levels are required for sperm motility, capacitation, the acrosome reaction, hyperactivation, and fertilization ability [27], so we can assume that ROS levels are inside of physiological range; however, further studies will be able to address the specific role of ROS in resveratrol’s effects in promoting a better spermatic performance. The strengths of our study include the use of a supplement with a highly bioavailable form of resveratrol (REVIFAST™) with an increased pharmacokinetic profile [34]. It can bridge the gap between the interesting in vitro effects that are otherwise not possible in vivo.

## 5. Conclusions

In conclusion, GENANTE^®^ improves the concentration and motility of sperm in idiopathic male infertility. This study also confirms that, taken together, the possibility of targeting the metabolic and energetic mechanisms involved in spermatogenesis and sperm motility with a promising molecule such as resveratrol could provide clinical benefits. However, a deeper understanding of the specific mechanisms involved is essential and further studies are needed to confirm our hypothesis.

## Figures and Tables

**Table 1:** The demographic and clinical characteristics of patients.

Patients	20
Age (mean ± SD)	30.9 ± 3.28
BMI (mean ± SD)	27.9 ± 1.4
Married *n* (%)	10 (50)
Erectile Dysfunction *n* (%)	0 (0)
Male hyopogonadism *n* (%)	0 (0)
Oligozoospermia *n* (%)	0 (0)
Asthenozoospermia *n* (%)	6 (30)
Teratozoospermia *n* (%)	7 (35)
Oligoasthenozoospermia *n* (%)	7 (95)
Oligoteratozoospermia *n* (%)	0 (0)
Asthenoteratozoospermia *n* (%)	0 (0)
Oligosthenoteratozoospermia *n* (%)	0 (0)
Normal scrotal ultrasound	20 (100)
Normal prostatic transrectal ultrasound	20 (100)
FSH mlU/mL (mean ± SD)	4.60 ± 1.3
LH mlU/mL (mean ± SD)	3.87 ± 1.8
Total Testosterone nmol/L (mean ± SD)	14.89 ± 0.3
Estradiol pg/mL (mean ± SD)	25.2 ± 2.1
Prolactin ng/mL (mean ± SD)	11.17 ± 1.9
25-OH-Vitamin D3 ng/mL (mean ± SD)	55.8 ± 2.8

FSH: follicle-stimulating hormone. LH: luteinizing hormone.

**Table 2:** Semen parameters at baseline 1, 3, and 6 months after recruitment.

Parameters	Baseline	1 Month	3 Months	6 Months	*p*-Value
Normal Viscosity *n* (%)	20 (100)	20 (100)	20(100)	20 (100)	nd
Complete Fludification *n* (%)	11(100)	11(100)	11(100)	11(100)	nd
PH (mean ± SD)	8.1 ± 0.1	8.0 ± 0.3	8.0 ± 0.2	8.0 ± 0.3	0.219
Semen volume (mL, mean ± SD)	3.6 ± 0.7	3.61 ± 0.6	3.61 ± 0.5	3.7 ± 0.5	0.525
Total sperm number (10^6^/ejaculate, mean ± SD)	41.5 ± 22.1	44.4 ± 22.4	47.7 ± 21.4	48.2 ± 22.4	0.002 *
Sperm concentration (10^6^/mL, mean ± SD)	22.6 ± 9.5	23.7 ±9.2	24.5 ± 9.1	25.7 ± 8.1	0.0001 *
Total motility (PR + NP, % mean ± SD)	48.3 ± 13.8	51.1 ± 12.8	55.7 ± 12.7	59.0 ± 12.8	0.0001 *
Progressive motility (PR > 32% mean ± SD)	31 ± 5.7	35.3 ± 7.1	40.2 ± 10.9	48.3 ± 11.2	0.0001 *
Sperm morphology (normal forms%)	14 (66.7)	14 (66.7)	14 (66.7)	14 (66.7)	nd

PR: progressive motility. NP: non-progressive motility. nd: not determined * (*p* ≤ 0.05).

**Table 3:** Hormonal evaluation at baseline 1, 3, and 6 months after recruitment.

Parameters	Baseline	1 Month	3 Months	6 Months	*p* Value
FSH mlU/mL (mean ± SD)	4.60 ± 1.3	4.62 ± 1.2	4.61 ± 1.5	4.61 ± 1.7	0.9
LH mlU/mL (mean ± SD)	3.87 ± 1.8	3.85 ± 1.3	3.87 ± 1.4	3.88 ± 1.4	0.87
Total Testosterone nmol/L (mean ± SD)	14.89 ± 0.3	14.84 ± 0.7	14.82 ± 0.2	14.87 ± 0.2	0.9
Estradiol pg/mL (mean ± SD)	25.2 ± 2.1	25.8 ± 2.4	25.5 ± 2.3	25.6 ± 2.0	0.86
Prolactin ng/mL (mean ± SD)	11.17 ± 1.9	11.13 ± 1.5	11.15 ± 1.3	11.19 ± 1.2	0.9
25-OH-Vitamin D3 ng/mL (mean ± SD)	55.8 ± 2.8	55.1 ± 2.2	55.7 ± 2.4	55.4 ± 2.1	0.9

FSH: follicle-stimulating hormone. LH: luteinizing hormone.

## Abbreviations

ADP	phosphorylate adenosine diphosphate
ATP	production of adenosine triphosphate
DP	mitochondrial membrane potential
ETC	electron transfer chain
FADH2	dihydroflavine-adenine dinucleotide
FOXOs	forkhead box transcription factor, class O
FSH	follicle-stimulating hormone
HSF1	heat shock factor 1
LH	luteinizing hormone
MTHFR	polymorphisms of the tetrahydrofolate methylene
NADH	nicotinamide adenine dinucleotide
NF-kB	kB gene binding nuclear factor
OXPHOS	oxidative phosphorylation
PPARγ	peroxisome proliferator-activated receptor γ
SIRT1	sirtuin 1
